# A Saga-In-Progress: Challenges and Milestones on Our Way Toward the *Nordic Core Values and Principles of Family Medicine/General Practice*

**DOI:** 10.3389/fmed.2021.681612

**Published:** 2021-11-26

**Authors:** Johann A. Sigurdsson, Anders Beich, Anna Stavdal

**Affiliations:** ^1^Nordic Federation of General Practice, Reykjavik, Iceland; ^2^Grafarvogur Health Care Center, Reykjavik, Iceland; ^3^Department of Public Health and Nursing/General Practice Research Unit, Norwegian University of Science and Technology (NTNU), Trondheim, Norway; ^4^Danish College of General Practice, Copenhagen, Denmark; ^5^Family Doctors Lietmann, Beich and Ehrenreich, Copenhagen, Denmark; ^6^World Organization of Family Doctors (WONCA), Oslo, Norway; ^7^Norwegian Research School in General Practice, Faculty of Medicine, Institute of Health and Society, University of Oslo, Oslo, Norway

**Keywords:** core values, sustainability, person-centered, continuity, history, family medicine

## Abstract

**Summary:** Late in 2020, the Nordic Colleges of General Practice published a joint statement specifying what General Practitioners stand for and intend to act upon, our *Core Values and Principles*. In this article, the authors describe and analyze challenges and milestones encountered on our 50-year journey toward the creation of that document.

The shaping of Family Medicine/General Practice as an academic discipline began in the 1960's. During an initial, descriptive phase, the new specialty was defined, its educational curricula formulated, and the core competencies required to earn the title, Specialist in Family Medicine, were identified. Focus was not yet placed directly on the relationship between viable working principles and values, however.

Then, the 1978 WHO *Alma Ata Declaration* affirmed health to be a fundamental human right, with primary health care as the heart of sustainable health care systems, indirectly mandating that the field of Family Medicine deliver value-based health care. A major step in that process was taken in 2001: The Norwegian College of General Practice launched their statement identifying the seven theses, *Sju teser*, that characterize the principles, purposes—and core values—of General Practice. Later, the Nordic colleges worked together to formulate the 2020 joint statement.

We are confident that Family Medicine will continue to provide sustainable, relationship-based care, and to protect the human side of medicine. Sharing *core values* and *principles* can help us mobilize as effective advocates for our discipline and for our patients, the citizens whom we serve.

## Introduction

Late in 2020, the Nordic Colleges of General Practice/Family Medicine, referred to here as “Family Medicine,” published their *Core Values and Principles* (1, 2). These are explicit statements of what we as General Practitioners (GPs) stand for and intend to act upon—the professional values as postulated by our colleges, and the individual, personal values and principles that are embedded within them. The outline of these statements is shown in Table 1 (see Supplementary Text for the full version, including values definitions).

**Table 1:** Core Values and Principles of Nordic General Practice/Family Medicine^*^.

**1**	We promote continuity of doctor-patient relationships as a central organizing principle.
**2**	We provide timely diagnosis and avoid unnecessary tests and overtreatment. Disease prevention and health promotion are integrated into our daily activities.
**3**	We prioritize those whose needs for healthcare are greatest.
**4**	We practice person-centered medicine, emphasizing dialogue, context, and the best evidence available.
**5**	We remain committed to education, research, and quality development.
**6**	We recognize that social strain, deprivation, and traumatic experiences increase people's susceptibility to disease, and we speak out on relevant issues.
**7**	We collaborate across professions and disciplines while also taking care not to blur the lines of responsibility.

* *Short version–from the Nordic Federation of General Practice. Scand J Prim Health Care. (2020) 38:367–8*.

### Societal Changes—Interest in Values Emerges

The focus of the pioneers developing Family Medicine in the mid-20th century was not primarily on the concept of values. Their extensive scholarly works explored: defining Family Medicine as a discipline; formulating educational curricula for these new generalists; debating which core competencies must be mastered to earn the title of “Specialist” in this new discipline; and envisioning the Family Medicine of the future. This was the crucial *descriptive* phase in the history of Family Medicine (further described in Supplementary Text).

During the 1990's, Family Medicine leaders from various countries began to examine *ethical issues*, questioning their aims, what to consider acceptable, what to deem worth fighting for, or against. Meantime, the profession had to adapt to changes within society and medicine, such as technological innovations and increasing commercialization. The fragmentation of care had also increased, leaving fewer stable communities, with more GPs working part-time while treating more patients, the emergence of “walk-in” centers and “screen doctoring” (3).

Inevitably, such developments challenged, perhaps even threatened, our profession, exposing an urgent need not merely to update it but to re-envision its very foundations. The European region's World Organization of Family Doctors, WONCA Europe, formulated a definition of Family Medicine (4) highlighting its essential concepts, its core professional competencies, and approaches to learning. This definition does not explicitly emphasize the relationship between viable working *principles* and *values*. Furthermore, while the terms *vision, mission*, and *values* were implicit within the earlier frameworks, they were not explored directly (2). Meantime, many terms that were assumed to be shared prove instead to have widely diverging definitions within the different languages, each carrying distinctive cultural implications and evoking unique—unshared—associations.

Ian McWhinney, considered the founding father of academic Family Medicine, described the principles of Family Medicine as early as 1981 as, “a distinctive worldview—a system of *values and an approach to problems—*that is identifiably different from that of other disciplines” (5). In 2002, Pendleton and King added, “If we are to release the potential motivating power of the vision and values in action, it may be time to articulate the values of our professions more explicitly” (6).

Words matter. Clearly, if the profession is to unify worldwide, GPs will need to agree on a *vision* and a set of *values*—based on a shared understanding of a common vocabulary. Carefully chosen words, slogans, and concepts have power when advocating for ideas and ideologies.

Thus, the aim of this paper is to chronicle the path that our community of GPs has taken during recent decades to investigate and shape our discipline, its fundamental mindset in general and its Nordic character in particular—with a special focus on *values*.

## Nordic Development of Family Medicine

**Figure 1:**
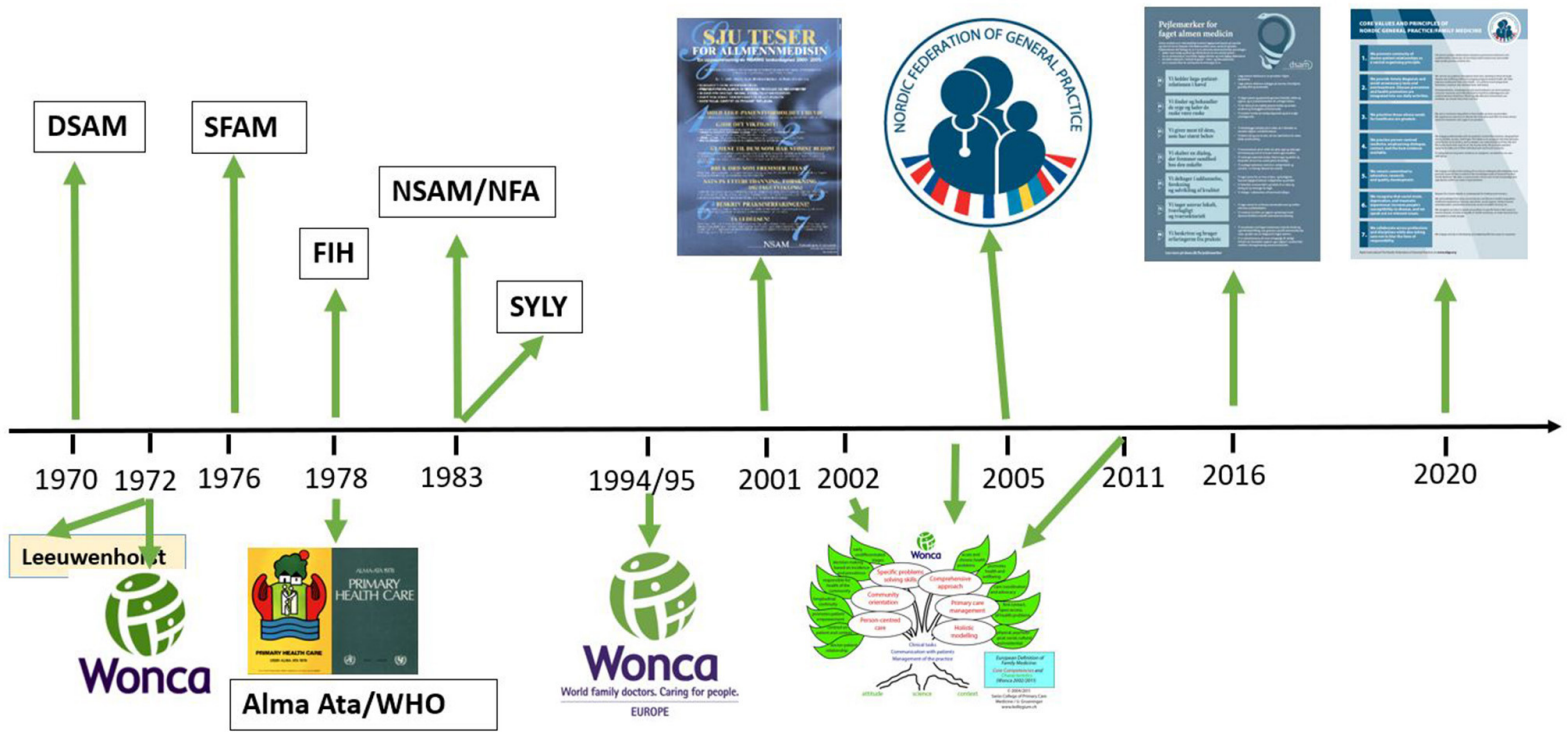
Milestones on our way toward the *Nordic core values and principles of general practice* (DSAM, Danish College of General Practice; Wonca, World Organization of Family Doctors; SFAM, Swedish College of General Practice; FIH, The Icelandic College of Family Physicians; NSAM/NFA, The Norwegian College of General Practice; SYLY, The Finnish Association of General Practice).

In the West, the rise of specialization combined with the increasing fragmentation of medical care that had begun by the 1950's to worsen the existing shortage of available GPs. In 1978, the declaration from the Alma Ata meeting emerged as a major milestone (7). At that meeting, international leaders declared that health is a fundamental human right with primary care being the key to the attainment of the goal of “Health for All”. The World Health Organization (WHO) soon adopted that view. This strengthened the GP's motivation to unite. Colleges of Family Medicine were established in the Nordic countries and elsewhere (Figure 1).

A new generation of pioneering doctors, mainly but not only in the United States and England, began emphasizing the need to teach Family Medicine in medical schools as a high-quality academic discipline. Modern Family Medicine, including the academic training of the “new generalists,” thus began to take shape (5). *The Graduate Education of Physicians* (8), known as the “Millis Report,” was among the first to specify curriculum requirements for this training, in a continuum extending through medical school, internship, and residency. It would take decades, however, to compress and clarify the vast contents and structure of the field of Family Medicine, as well as to develop appropriate vocational training programs (These historical processes are described in the Supplementary Text).

### The Risk Factor Paradigm

During the 1960's, cardiovascular epidemiological studies, such as the famous Framingham study, increased in number worldwide, and had a considerable impact on the development of Family Medicine and its values as described below. These studies documented high rates of cardiovascular diseases (CVD) associated with elevated blood pressure, high cholesterol, and smoking, which were then identified as disease risk factors (9, 10). Risk factors became equivalent to disease definitions. These important findings had a great impact on the *curative* as well as *predictive-preventive* medical interventions that were supported by governments and WHO.

Advanced statistics were also introduced during this period, with “Significant difference,” as illustrated in *p-values*, playing a major role. The best evidence, defined as *p* < 0.001, soon became a “golden goose,” marking the arrival of the *Risk Factor Paradigm* (9). CVD risk factor studies became quite popular during the late 20th century. Also during that period, a trend emerged to expand disease definitions, resulting in more and more people who had previously been considered healthy now being given a diagnosis, thus labeled as sick. When follow-up intervention studies failed to yield convincing results, larger studies were designed to ensure reaching the *p* < 0.001 level—that is, to demonstrate significant differences between those “at risk” vs. those “not at risk”. As the size of these studies increased, their results became less relevant to clinical settings. Nonetheless, healthy people continued to be promised even better health than they already enjoyed.

It also bears mentioning that most of the big intervention studies were designed and conducted to evaluate new types of drugs, and thus were initiated, and financed, by the pharmaceutical industry.

#### The Process in Iceland

As the academic discipline and specialty of Family Medicine began taking shape in the 1960's, the interest in it grew. The new Icelandic Primary Health Care legislation of 1974 mandated the building of primary health care centers in every county, staffed by well-educated GPs, nurses, midwives, secretaries, and ancillary staff. Customarily, most Icelandic doctors completed their postgraduate education abroad. The advanced Family Medicine specialization programs in Canada had come farthest, offering training based on critical thinking, and practice conducting video-guided Family Medicine consultations (11). Our Icelandic pioneer, Olafur Mixa, GP, graduated from Calgary in 1971, becoming the first Nordic Specialist in Family Medicine to be educated according to the new curriculum of professional training; he was also an early advocate for these programs. A group of young, enthusiastic Icelandic doctors, fascinated by such ideas and opportunities, soon enrolled in the Canadian programs. Their teachers included Ian R. McWhinney (Western University) and David Sackett (McMaster). While not famous then, they would soon be recognized as the vanguard of examining, and thinking critically about, the ideology and *principles* of the medical establishment. The “bible” for our young doctors at that time was Ivan Illich's*, Limits to Medicine* (12), understood in retrospect as being filled with reflections on *values*. Furthermore, Sackett and his co-workers managed to popularize their course, *Critical Appraisal of the Medical Literature*, later entitled, *Evidence Based Medicine*.

### Fragmentation of Care and a Territorial Battle

The young Icelandic GPs returning to Iceland early in the 1980's after completing their specialty training in Canada, Sweden, and England, were full of fervor. They were (at least according to themselves) exceedingly well-versed not only in CVD epidemiology but also critical thinking and the ambitious ideology of Family Medicine—including its *vision*.

*Fragmentation of care*, however, had already re-shaped the Primary Care landscape in Iceland, with baby wellness care and school health care delegated to Pediatricians, maternity care placed within the domain of Gynecologists, etc. No wonder the new Family Medicine discipline, with all its *principles* and purposes, sparked both debate and conflict.

As did most Western countries, Iceland ran a comprehensive, national, epidemiological CVD study; they began in 1967, under the auspices of the Icelandic Heart Association. In addition to carrying out a study of CVD risk factors on carefully selected cohorts, the Heart Association offered all Icelandic citizens the opportunity to receive such “health checks” as funding came through the Social Security system, they could participate almost free of charge. These health checks were greatly appreciated and very popular (13). The notion that “More is Better” took hold, with ever more people being convinced that the strategy of “running all tests known to man—and as soon as possible” would improve their health.

Younger GPs began to object, however. They argued that such comprehensive “screenings” were unethical, did not meet generally accepted screening criteria, fell outside the framework of research, and, in addition, jeopardized the *value* of offering a holistic approach, as well as the new plans that Primary Health Care would serve as the entry point for medical contact. The debate culminated in 1983. The Ethical Committee of the Icelandic Medical Association concluded that it had been the critique put forth by the younger GPs that had not only been unethical but had also exemplified unacceptable collegial behavior (13)!

Thus, the debate that emerged between GPs and the heads of the Icelandic Heart Association became a battle about the *values* embedded in Evidence Based Medicine.

#### The Process in Norway

In the mid-1980's, Jostein Holmen, GP, then the upcoming leader of the epidemiological HUNT study and later Professor of Community Medicine, began to critique the clinical guidelines for treating high blood pressure, in particular the cut-off levels recommended by CVD cardiologists (14, 15). He and his co-workers argued that “reductionist” organ specialists needed only to focus on one disease or condition (later referred to as “linear thinking”), independent of other levels of “risk.” GPs, on the other hand, and their colleagues within community medicine, had to respond not merely to patient's needs but also to such concerns as public funding and the equitable distribution of services (15). This aspect of the development of Family Medicine would be elaborated later, relating it to non-linear, complex, adaptive systems theories (16).

### Is Risk a Disease?

The *Risk Project* was formally established in 1994 by the Norwegian College of General Practice (NSAM, later NFA), under the leadership of Elisabeth Swensen, GP, and with the support of the Norwegian Ministry of Health. The topic of *values* within Family Medicine vs. those of other disciplines was explored in, *Diagnose: Risiko* (*Diagnosis: At Risk)*, edited by Swensen (17), using events of 1989 as a case-in-point. In that year, all Norwegian citizens aged 40–42 residing in various counties were invited to participate in a comprehensive population study of cardiovascular risk factors (18). Arranged by The Norwegian Center for Health Research (Statens helseundersøkelser, SHUS), the study was promoted through an intensive information campaign in the mass media and elsewhere, presenting the benefits of such preventive measures as, “An offer you can't refuse” (17).

Soon, however, it became clear that the plan involved delegating much of the burden of carrying out years of follow-up work to the local GPs. Those with well-established practices grasped immediately that such added obligations would not glide smoothly into everyday General Practice clinical life. Some resistance—and anger—began to manifest within NSAM. In Norway, as in Iceland, the GP's clear, albeit unspoken, message was simple: “You are welcome to run your own shop—but not inside my shop.”

### A Norwegian “Critical Mass” Is Reached

Questions arose: Who would define the problem? What sources of knowledge would be deemed valid, and by whose authority? What consequences would result? What actual benefits, if any, might this intervention yield?

Some GPs had raised such issues earlier, at the very start of the study, but without awakening much interest among health authorities and researchers (17). However, when the number of people posing this sort of question finally reached a critical mass, a common objective emerged: to examine evidence that sheds light on the discipline of Family Medicine in general, and on the role of the GP in particular.

### The Seven Principles of Family Medicine/General Practice

Discussions and debates at the NSAM meetings in Norway, began to focus on what GPs stand for and want to prioritize. Based on this work, and under the leadership of Anna Stavdal, GP, NSAM drew up their *Sju teser* (*Seven Principles of Good Medical Practice for General Practitioners*). In 2001, this itemized, passionate, expression (in Norwegian) of GP's *professional values and principles* was published and distributed, formatted as an eye-catching poster (Figure 1) (1, 19–21). Here, the seven main principles are specified as imperatives, with clarifications of relevant principles and actions next to each. We have seen since then what an effective tool for advocating for our profession and our patients that this short, striking poster has proven to be.

The 2001 English translation draft of Principle 2. of the *Sju teser* reads: “Do what is most important,” further clarified by, “prioritizing patients with conditions in need of treatment, and sparing patients from wrongfully being treated as sick.” Today, we might have written, “avoid overmedicalization and overtreatment,” but those terms had not yet entered the medical discourse.

The ongoing debates in the Nordic countries, including those regarding Principle 2., convinced Irene Hetlevik, GP, later Professor of Family Medicine, and Stavdal, of the need to adapt the Norwegian *Sju teser* to suit the Nordic milieu. In 2004, they established the *Nordic Risk Group* (NRG), under Hetlevik's leadership (22). Inspired by such industrial companies as Toyota, the group began to refer to its tasks and actions in terms of *vision* and *mission*. As far as we know, this was the first time those concepts were applied in the context of Nordic Family Medicine. The NRG saw *value* as being implicit within the concept of *vision*. *Medicalization*, which had entered the discussions regarding good medical practice by then, was also seen as being incorporated within *vision*, as in, “… systematically aiming to minimize medicalization and risk-labeling” (22, 23). Later, the campaign against unnecessary health care interventions called “Choosing Wisely” came on the scene in the Nordic countries and elsewhere.

#### The Process in Sweden

Within the Nordic countries, the pace of implementing modern Family Medicine has varied, and that saga can not be told fully here. While some of our colleagues were fighting for Family Medicine to serve as an organizing concept, others formed groups to focus on specific topics. In Sweden, the ideology of Family Medicine was met with great resistance, primarily from representatives of various specialties. In the 1980's, two prominent colleagues, Göran Sjönell, GP, later President of WONCA, and Carl Edward Rudebeck, GP, later Professor of Family Medicine, initiated a heroic fight for modern Family Medicine (3, 24). In 1988, when Sjönell and colleagues cast doubts on the evidence used to justify routine, breast cancer screening using mammography, a heated and longstanding debate was ignited (25, 26). Our Swedish colleague's experiences contribute significant insight and support to the development of our common Nordic Core Values.

#### The Process in Denmark

Most of the above processes were ongoing in other parts of the world as well, and were interconnected. In Denmark, in the late 1990's, Hanne Hollnagel, GP, Professor of Family Medicine and head researcher for the Glostrup Epidemiological Study, took the initiative to organize debates on the risk concept, similar to those in which our colleagues in Norway, Sweden, and Iceland were engaged (27, 28).

### Investing in General Participation and *Personal values*

In 1998, Carl Erik Mabeck, GP, Professor of Family Medicine, led Danish GPs in arranging a workshop to explore the future of General Practice (*Et fremtidsværksted)*. Implicit in the title of their published brochure, *Discussion Paper on Core Functions of General Practice* (29), is an acknowledgment that ongoing discussions regarding the core functions of General Practice are both important and necessary. It also indicates support for the type of self-awareness the Balint model includes, one which gradually leads to a “…limited but substantial change in the GP's personality” (30).

In 2014, the Organization of General Practitioners in Denmark (PLO) joined with the Danish College (DSAM), to launch an extensive *vision* process. They collected data from hundreds of Danish GPs participating in nation-wide focus group discussions, who had agreed to share their views on the *core principles* and purposes of General Practice. Although political concerns put a stop to their *vision* process, the organizers did not waste their collected data. In 2016, inspired by the structure of the Norwegian *Sju teser* of 2001, DSAM, headed by one of the authors here (AB), utilized analyses of that data to help them formulate *Pejlemaerker for faget almen medicin* (*Guideposts for the Profession of General Practice Medicine*), their own, updated version of their professional *principles* and *values*. Instead of phrasing these as seven imperatives, they expressed them as *personal values* by using first-person plural descriptions: “We do….” (2, 31).

#### The Nordic Federation's Process

Finland, Sweden, Norway, Denmark, and Iceland have been collaborating productively for decades (32–34). They share common history, culture, and ideology, as well as knowledge, skills, and attitudes, regarding Family Medicine, their Nordic colleagues, and their academic institutions (32). In 2005, this collaboration was formalized through the establishment of the Nordic Federation of General Practice (NFGP) (35).

In 2017, the Nordic Federation decided to reexamine our tasks and objectives, ultimately expressed as *vision* and *mission* statements. The first aim of this project was to merge the Norwegian *Sju teser* and the Danish *Pejlemaerker for faget almen medicin* into an English-language statement on which we could all agree. By 2020, we had our: *Core Values and Principles of Nordic Family Medicine*. The second project aim was to reach out digitally to our Nordic networks, associations, and congresses, to engage as many Nordic colleagues as possible in a shared awareness process. The third aim was to devise ways to adapt and extend our own consensus process to include colleagues and their associations in countries beyond our own region. These processes have been described more fully elsewhere (2).

## Strengths and Limitations of This Analysis

The main strength of our analysis of this Saga-In-Progress lies in the fact that we, the authors, not only experienced the fifty years covered here as active GPs but were also opinion leaders in the Nordic evolution of modern Family Medicine. Those years of the new discipline's development were often turbulent, with hefty debates going on within the health care establishment. Consequently, the main limitation of this analysis may be that it is undeniably from our perspective.

## Epilogue and Conclusions

The journey toward formulating our *core values* that we have documented here may also be understood in terms of the WHO *Alma Ata Declaration* of 1978 mentioned above. Viewed from that perspective, the WHO did indeed mandate the field of Family Medicine to provide value-based health care. As stated in the *Alma Ata Declaration*:

…The Conference strongly reaffirms that health, which is a state of complete physical, mental and social well-being, and not merely the absence of disease or infirmity, is a fundamental human right and that the attainment of the highest possible level of health is a most important world-wide social goal whose realization requires the action of many other social and economic sectors in addition to the health sector.

GPs and stakeholders of Family Medicine have acted accordingly. A substantial body of research and literature described elsewhere confirms the relevance and validity of Family Medicine, associating it with better health, better health care, and lower costs (2, 36–39). Among the conclusions to be drawn from the history above is that, to understand General Practice, knowledge must be combined with a specific skill set and a particular attitude. In other words, implementing a holistic approach involves more than addressing the complexity of patient's problems; it also requires an understanding of the complexity of the role of Family Medicine within society.

Although our *values* will endure, standards of care do indeed vary, depending on the economy, organization, and/or political situation of each country. As the WHO *Alma Ata Declaration* acknowledges, standards of care must be tailored to what the community/country can afford. Consequently, the precise formulations of the *Core Values and Principles* that the Nordic Colleges have put forth can not, and should not, be assumed to be applicable everywhere; each culture/country must consider what adaptations are needed. Nonetheless, our *Nordic Core Values and Principles* statement, and our process of arriving at it, serve as useful resources.

In a recent paper published in this journal, Arvidsson et al. (40) recount the ongoing discussions about European *core values* and the challenges being faced. They note that the attention given to these concepts is increasing as processes to define *core values* emerge in various European countries.

In line with the WHO's declaration regarding primary health care (7, 41, 42), Family Medicine will continue to provide sustainable, responsible, relationship-based care, and to protect the human side of medicine. Meantime, as described earlier, our organizational principles and clinical priorities are challenged, repeatedly, by stakeholders outside our discipline. In such situations, awareness processes among our colleagues, colleges, and our WONCA family are of utmost importance. Sharing a common set of c*ore values and principles* can motivate and mobilize us as advocates both for our discipline and for our patients, the citizens whom we serve.

## Data Availability Statement

The raw data supporting the conclusions of this article will be made available by the authors, without undue reservation.

## Author Contributions

JS and AS: conceived of the idea for this paper. JS, AB, and AS: contributed to its planning. JS: wrote the first draft. All authors: read and approved the final manuscript.

## Funding

The Research Fund of the Icelandic College of Family Physicians and the Nordic Federation of General Practice contributed to this work.

## Conflict of Interest

The authors declare that the research was conducted in the absence of any commercial or financial relationships that could be construed as a potential conflict of interest.

## Publisher's Note

All claims expressed in this article are solely those of the authors and do not necessarily represent those of their affiliated organizations, or those of the publisher, the editors and the reviewers. Any product that may be evaluated in this article, or claim that may be made by its manufacturer, is not guaranteed or endorsed by the publisher.
