# Supplementary material for: A Saga-In-Progress: Challenges and Milestones on Our Way Toward the Nordic Core Values and Principles of Family Medicine/General Practice
Source: Front Med (Lausanne). 2021 Nov 26;8:681612. doi: 10.3389/fmed.2021.681612 (PMC8662748; doi:10.3389/fmed.2021.681612)
Supplement: Supplementary file 1 [file Table_1.DOCX]

**SUPPLEMENTARY TEXT**

| **Table 1. Core Values and Principles of Nordic General Practice/Family Medicine*** | |
| --- | --- |
| [WHO](https://www.who.int/docs/default-source/primary-health/declaration/gcphc-declaration.pdf) considers primary health care to be a cornerstone of sustainable health care systems. General Practice/Family Medicine is the key provider of primary health care.  [WONCA Europe](https://www.woncaeurope.org/file/520e8ed3-30b4-4a74-bc35-87286d3de5c7/Definition%203rd%20ed%202011%20with%20revised%20wonca%20tree.pdf) has defined General Practice/Family Medicine as both a clinical specialty and a discipline in its own right, with its own distinct curriculum and research base. As an academic discipline, General Practice/Family Medicine is based on knowledge and methodology drawn from the Natural Sciences as well as the Humanities.  ***As committed leaders*** *in the ongoing process of defining and implementing core values and principles, General Practitioners aim to:*  - promote and protect the health and well-being of each individual patient while keeping in mind the needs of the general population;  - provide a frame of reference for our professional identity;  - provide a basis for continuing professional development, with curricula and training adapted to every educational level – undergraduate, post-graduate, and beyond;  - communicate our mandate and the principles of our work to patients, fellow healthcare workers, and the communities we serve. | |
| **1** | **We promote continuity of doctor-patient relationships as a central organising principle.**  The doctor-patient relationship is based on personal involvement and confidentiality. Continuity of care helps build mutual trust and enable high-quality, person-centred care. |
| **2** | **We provide timely diagnosis and avoid unnecessary tests and overtreatment. Disease prevention and health promotion are integrated into our daily activities.**  We care for our patients throughout their lives, tending to them through disease and suffering while encouraging progress toward health. We help patients understand their own health – to confront and manage their limitations, improve and maintain their well-being.  Overexamination, overdiagnosis, and overtreatment can harm patients, consume resources, and indirectly lead to harmful underdiagnosis and undertreatment elsewhere. When equally effective interventions are available, we choose those that cost less. |
| **3** | **We prioritise those whose needs for healthcare are greatest.**  We aim to minimise inequalities in how health services are provided. We organise our practices to devote the most time and effort to those whose needs for treatment and support are greatest. |
| **4** | **We practice person-centred medicine, emphasising dialogue, context, and the best evidence available.**  We engage professionally with our patients’ current life situations, biographical stories, beliefs, worries, and hopes. This helps us to recognise the links between social factors and sickness, and to deepen our understanding of how life and life events leave their imprint on the human body. We promote patients’ capacity to make use of their individual and communal resources.  To safeguard our long-term resilience as caregivers, we attend to our own well-being. |
| **5** | **We remain committed to education, research, and quality development.**  We engage actively in the training of our future colleagues. We implement and promote research that is suited to the knowledge needs of General Practice/Family Medicine. We take a constructively critical view of new knowledge and approaches within our areas of specialisation. |
| **6** | **We recognise that social strain, deprivation, and traumatic experiences increase people’s susceptibility to disease, and we speak out on relevant issues.**  Respect for human dignity is a prerequisite for healing and recovery.  We acknowledge that many circumstances contribute to health inequalities: childhood experiences, housing, education, social support, family income/ unemployment, community structures, access to health services, etc.  We recognise our duty to speak out publicly on specific factors that cause or worsen disease, increase inequality in health outcomes, or make resources less accessible to certain people. |
| **7** | **We collaborate across professions and disciplines while also taking care not to blur the lines of responsibility.**  We engage actively in developing and adapting effective ways to cooperate. |

*<https://www.nfgp.org/>

**The Authors’ Perspective**

Having entered the developmental process of modern Family Medicine (FM) early – during the 1970’s – we, the GP authors, belong to the 2^nd^ and 3^rd^ generations of its evolution (1). This story shares our perspective on some of the challenges and milestones we’ve witnessed on our way. Of course, no journey starts at some point zero; other explorers have long ago walked the paths we follow. In retrospect, it may seem obvious that Family Medicine/General Practice has been evolving worldwide, leaving traces of what we may now recognize as feedback loops, spirals of step-by-step discussions. Earlier on, however, such connections were not nearly so obvious.

**Why Values? Some Definitions**

Although health care systems do not endorse any single definition, *values* are embedded into the profession’s vision, both of what we strive toward, and of the world as we believe it ought to be (2). Some *values* are deemed *universal*, such as compassion, respect, justice, equity, and integrity (3). Others are considered *personal*, serving as guidelines for individual behavior and choices. *Professional values*, meanwhile, serve to guide a discipline’s members as a group. Such values may be selected, carefully, to suit each profession, shaping the group’s identity, principles, and beliefs. Generally, these values are implicit within the profession’s core ethics (4). By the time individuals have qualified to assume their professional role, a strong relationship has usually – although not always – developed between their *personal* and *professional values*.

Traditions impact values, such as those regarding doctor-patient relationships (5). It may be unnecessary to describe the fundamental values of a particular profession explicitly in countries where basic values, such as equity and justice, are already embedded into the political, legal, and cultural commitments. In countries where such basic values are absent, whether from their legal system, their traditions, or both, medical professionals (among others) may choose to prioritize – explicitly and overtly – the values that they consider essential to their professionalism.

Pendleton and King point out that the basis for trust is laid when core values are both declared and acted upon; if, however, they are stated but not adhered to, whatever trust may exist becomes damaged.

Statements of values indicate what is important; statements of professional or organizational standards define what to consider as good or acceptable (6).

**Organizational Development of Networks of European Associations**

In Europe, the U.K.’s Royal College of General Practice (RCGP) took the lead in describing the new discipline of Family Medicine. In 1972, a working party there, led by John Horder, published, *The Future General Practitioner: Learning and Teaching* (7).

However, the job of the ‘new generalist’ remained to be defined. That same year saw the start of the European Family Medicine collaboration. The Dutch College of General Practitioners convened a meeting at Leeuwenhorst, Holland, attended by 14 representatives from 11 European countries, to discuss the European General Practitioner of the future. At that meeting, John Horder joined Niels Bentzen (Denmark) in suggesting that further work be done, and the Leeuwenhorst Group was established (8). Horder describes in his memoir, *An Account of My Life*, how, during his earlier participation in the first U.K. Balint group, he became convinced of the importance the doctor-patient relationship has in a consultation (9). In 1974, the Leeuwenhorst Group published the oft-cited, *General Practitioner in Europe: A statement by the working party appointed by the second European conference on the Teaching of General Practice* (10), with Niels Bentzen and Christian F. Borchgrevink (Norway) as our Nordic representatives. Their main emphases were on describing the work of the GP and on detailing educational prerequisites: GPs should provide comprehensive, personal, primary, and continuous care; illness and disease should be regarded within a holistic context, with physical, psychological, and social factors taken into consideration; GPs’ education must equip them to fulfill precisely those defined requirements.

During the 1980’s, as the original Leeuwenhorst Group founders retired, the 22-member New Leeuwenhorst Group was established. The Nordic representatives were Carl Edvard Rudebeck (Sweden), Mårten Kvist (Finland), Ludvik Olafsson (Iceland), Dag H. Søevik (Norway), and Ib Svendsen (Denmark) (11). The new group continued to work on defining the tasks of Family Medicine (11).

At a 1994 meeting in Portugal, members of WONCA and the Italian SIMG decided to merge the two organizations, forming the European Society of General Practice/Family Medicine. WONCA Europe was inaugurated in 1995, at the European Parliament in Strasbourg, France. It gathered the existing European network of organizations under its umbrella (see the WONCA Europe homepage: <http://www.woncaeurope.org>).

In 2002, WONCA Europe published their first European definitions of General Practice (12). Revised in 2005 and again in 2011, its full title is: *European definitions of the key features of the discipline of General Practice, the role of the General Practitioner, and a description of the core competencies of General Practitioner/Family Physician*. (The 2005 edition was the first to include the now well-known WONCA tree illustration, which the Swiss College of Primary Care Medicine had created.)

That paper lays out, in English, the common understanding that the European WONCA members had arrived at regarding the characteristics of our discipline, as well as the core competencies each GP must master. All its aspects are based on best available evidence and take both context and attitude into account. Looking back, it seems clear that EURACT activities came to reflect perspectives that the Leeuwenhorst groups had developed.

**REFERENCES**

1. Sigurdsson JA, Beich A, Stavdal A. Our core values will endure. *Scand J Prim Health Care***.** 2020;38(4):363-6. //doi.org/10.1080/02813432.2020.1842676.
2. Kelly MP, Heath I, Howick J, Greenhalgh T. The importance of values in evidence-based medicine. *BMC Medical Ethics.* 2015;16:69.
3. [Rider](https://pubmed.ncbi.nlm.nih.gov/?term=Rider+EA&cauthor_id=25103181) EA, [Kurtz](https://pubmed.ncbi.nlm.nih.gov/?term=Kurtz+S&cauthor_id=25103181) S, [Slade](https://pubmed.ncbi.nlm.nih.gov/?term=Slade+D&cauthor_id=25103181) D, [Longmaid III](https://pubmed.ncbi.nlm.nih.gov/?term=Longmaid+HE+3rd&cauthor_id=25103181) HE, [Ming-Jung Ho](https://pubmed.ncbi.nlm.nih.gov/?term=Ho+MJ&cauthor_id=25103181), [Jack Kwok-hung Pun](https://pubmed.ncbi.nlm.nih.gov/?term=Pun+JK&cauthor_id=25103181) et al. The International Charter for Human Values in Healthcare: An interprofessional global collaboration to enhance values and communication in healthcare. *Patient Educ Couns*. 2014;96(3):273-80.
4. Moyo M, Goodyear-Smith FA, Weller J, Robb G, Shulruf B. Healthcare practitioners´ personal and professional values. *Adv in Health Sci Edu*. 2016;21:257-86.
5. McWhinney IR. Primary care: core values in a changing world. *BMJ*.1998;316:1807-9.
6. Pendleton D, King J. Values and leadership. *BMJ*.2002;325:1352-55.
7. Horder J, Byrne P, Freeling P, Harris C, Irvine D, Marinker M. Editors, Working Party of Royal College of General Practitioners (RCGP). Book: *The Future General Practitioner. Learning and Teaching*. London: BMJ, B.M.A. House, Tavistock Square; 1972. 265.
8. Horder J. An account of my life. *London J Prim Care*. 2011;4:79-82.
9. Horder J. An account of my life. *London J Prim Care.* 2010;3:69-61.
10. Leeuwenhorst Group, Netherlands 1974; available at: <https://euract.woncaeurope.org/sites/euractdev/files/documents/archive/publications/general-practitioner-europe-statement-working-party-appointed-2nd-european-conference-teaching.pdf> (accessed March 2021).
11. The New Leeuwenhorst Group. A European working party aiming to promote General Practice as a discipline for learning and teaching, 1985; available at:

<https://euract.woncaeurope.org/sites/euractdev/files/documents/archive/publications/commentary-present-state-learning-and-teaching-general-practice-europe-new-leeuwenhorst-group-1985.pdf> (accessed January 2021).

1. WOCNA Europe. The European definition of General Practice/Family Medicine 2^nd^ ed. 2011. <http://www.globalfamilydoctor.com/site/DefaultSite/filesystem/documents/regionDocs/European%20Definition%20of%20general%20practice%203rd%20ed%202011.pdf>.
